# Improved Image Quality for Static BLADE Magnetic Resonance Imaging Using the Total-Variation Regularized Least Absolute Deviation Solver

**DOI:** 10.3390/tomography7040048

**Published:** 2021-10-08

**Authors:** Hsin-Chia Chen, Haw-Chiao Yang, Chih-Ching Chen, Seb Harrevelt, Yu-Chieh Chao, Jyh-Miin Lin, Wei-Hsuan Yu, Hing-Chiu Chang, Chin-Kuo Chang, Feng-Nan Hwang

**Affiliations:** 1 Department of Diagnostic Medical Imaging, Madou Sin-Lau Hospital, Tainan 721, Taiwan; D4806@sinlau.org.tw (H.-C.C.); D4805@sinlau.org.tw (H.-C.Y.); slh182@sinlau.org.tw (Y.-C.C.); 2 Department of Finance, Chung Yuan Christian University, Chung Li 320, Taiwan; d97244002@ntu.edu.tw; 3 Department of Biomedical Engineering, Eindhoven University of Technology, 5612 AZ Eindhoven, The Netherlands; s.d.harrevelt@tue.nl; 4 Development and Alumni Relations, University of Cambridge, Cambridge CB5 8AB, UK; 5 Department of Mathematics, National Central University, Taoyuan City 320, Taiwan; whyu@math.ncu.edu.tw (W.-H.Y.); hwangf@math.ncu.edu.tw (F.-N.H.); 6 Department of Biomedical Engineering, The Chinese University of Hong Kong, Hong Kong; d98945001@ntu.edu.tw; 7 Global Health Program, College of Public Health, National Taiwan University, Taipei City 100, Taiwan; chinkuochang@ntu.edu.tw; 8 Institute of Epidemiology and Preventive Medicine, College of Public Health, National Taiwan University, Taipei City 100, Taiwan; 9 Institute of Psychiatry, Psychology, and Neuroscience, King’s College London, London SE5 8AF, UK

**Keywords:** BLADE MRI, least absolute deviation, graphic processing unit (GPU), non-uniform fast Fourier transform (NUFFT)

## Abstract

In order to improve the image quality of BLADE magnetic resonance imaging (MRI) using the index tensor solvers and to evaluate MRI image quality in a clinical setting, we implemented BLADE MRI reconstructions using two tensor solvers (the least-squares solver and the L1 total-variation regularized least absolute deviation (L1TV-LAD) solver) on a graphics processing unit (GPU). The BLADE raw data were prospectively acquired and presented in random order before being assessed by two independent radiologists. Evaluation scores were examined for consistency and then by repeated measures analysis of variance (ANOVA) to identify the superior algorithm. The simulation showed the structural similarity index (SSIM) of various tensor solvers ranged between 0.995 and 0.999. Inter-reader reliability was high (Intraclass correlation coefficient (ICC) = 0.845, 95% confidence interval: 0.817, 0.87). The image score of L1TV-LAD was significantly higher than that of vendor-provided image and the least-squares method. The image score of the least-squares method was significantly lower than that of the vendor-provided image. No significance was identified in L1TV-LAD with a regularization strength of λ= 0.4–1.0. The L1TV-LAD with a regularization strength of λ= 0.4–0.7 was found consistently better than least-squares and vendor-provided reconstruction in BLADE MRI with a SENSitivity Encoding (SENSE) factor of 2. This warrants further development of the integrated computing system with the scanner.

## 1. Introduction

The BLADE sequence (also known as the Periodically Rotated Overlapping ParallEL Lines with Enhanced Reconstruction (PROPELLER) magnetic resonance imaging (MRI) [1] or MultiVane) uses multiple overlapping rectangular k-space patches (the Fourier domain), which cover the circular region in k-space while sharing the k-space center. It is well known that BLADE MRI reduces the motion artifacts and assists the scanning process if patients are uncooperative. The motion insensitivity of BLADE MRI is achieved by retrospective correction for translational and rotational motion. For instance, BLADE MRI is effective in reducing motion artifact [2,3,4,5], which is useful in brain, spine, pediatric [6] and abdominal imaging. Recently, BLADE MRI has been applied to detect pulmonary damages related to coronavirus disease 2019 (COVID-19) [7]. All of these clinical applications reveal the value of BLADE MRI for imaging regions in motion, but its role in static imaging remains uncertain without more investigation.

Previously, BLADE MRI was noted to exhibit less sharp imagery than Cartesian k-space acquisition [8,9], as a result of using a vendor-provided reconstruction algorithm. Thus, BLADE MRI is usually chosen as an extra second pulse sequence only when motion artifacts drastically degrade the image quality. In our imaging facility, we aim to adopt BLADE MRI as a routine examination in cooperative patients, but the reconstruction method an be improved to sharpen the imagery. The clinically available protocol modifies the overlapping blades around the k-space center by a interleaved pattern with a SENSitivity Encoding (SENSE) acceleration factor of 2. It is known that SENSE acceleration halves the total scanning time from 150% (compared to the fully sampled turbo spin-echo) to 75%, but it causes much noise [10]. Image denoising using regularization is one way of mitigating the noise effect.

However, image reconstruction of the non-Cartesian data has been challenging, since no simple direct inverse transform is available. Many regularization methods have been proposed. For instance, over-complete sparse transforms, i.e., the stationary wavelet transforms, may be implemented to overcome the common blocky or oil-painting artifacts, but it requires significant effort to implement the complex algorithms. Recent optimization algorithms could be used to solve L1 minimization problems, e.g., the alternating direction method of multipliers (ADMM) [11], the fast iterative shrinkage-thresholding algorithm (FISTA) [12], or the proximal optimal gradient method (POGM) [13], which can obtain improved image quality at faster convergence rates. All of these techniques, however, may introduce additional complexities into the process of image reconstruction, thereby slowing down its clinical adoption.

Apart from recent dedicated regularization methods, image reconstruction using efficient total-variation (TV) regularization is obstructed by concerns about the suitability of TV for clinical MRI. Apparent stair-casing or oil-painting artifacts have been identified in TV regularization [14,15]. However, recent studies have shown that the disadvantages of TV-related artifacts can be mitigated by using least absolute deviation (LAD) data fidelity [16,17,18,19,20] as opposed to common ordinary least squares (OLS) data fidelity. A well characterized property of LAD is its robustness to outliers and sampling noise, and LAD has been applied to image interpolation [21], linear regression [22,23,24], neural networks [25], and the problem of regularized image restoration [22], but its application to MRI reconstruction requires further evaluation. It is worth noting that the integration of LAD and TV may generate an efficient algorithm for clinical purpose.

Furthermore, a crucial factor in parallel imaging is the smoothness of the coil estimation methods, which greatly influence the quality of the complex-valued imagery. Phase singularity has appeared in the recent literature, such as deep-learning frameworks [26] or phase-contrast MRI [27], forcing one to include the second dominant eigenvector [28]. Some networks effectively mitigate the phase singularity of eigen-decomposition [29,30]. Another approach is to leverage the phase unwrapping algorithms [31] to address the sharp phase transition in coil sensitivities, which may prove useful in future clinical applications. Recently deep-learning-based MRI reconstruction has solved the parallel image reconstruction problem with deep-SENSE [32] and GRAPPAnet [33].

This study is a continuing effort to bridge the gap between recent methodological developments [34,35] and image quality assessment in a clinical setting. In this study, we tested the clinical value of the L1TV-LAD method on the GPU, which is immune to the stair-casing artifacts of TV. In addition, a new coil estimation method was integrated into the reconstruction pipeline without phase singularity. In the present study, the tensor iteration generates smooth coil sensitivities, which appear to be more homogeneous than that obtained with the power iteration or the root-sum-squared (RSS) method. The tensor iterations naturally impose a low-frequency constraint, and phase singularity is mitigated. Our method is conceptually akin to the simultaneous estimation for imagery and coil sensitivity [36], although we consider its index tensor form without including the Sobolev-norm weight [37]. We tested the image quality of the L1TV-LAD reconstruction in a clinical setting in order to assess the value of the algorithm. We discuss the implications of this study and the outlook for future studies.

## 2. Materials and Methods

### 2.1. Mathematical Background

Index tensor notation has provided a handy tool for annotating multi-dimensional operators beyond second order matrices. The adoption of the multi-dimensional indexed tensors is useful to the non-Cartesian MRI acquisition modes. In this study, we investigated the index tensor notation for non-Cartesian BLADE MRI encoding method. The technical details are outlined in Appendix A.

### 2.2. Reconstruction of In Vivo Rawdata

**Figure 1:**
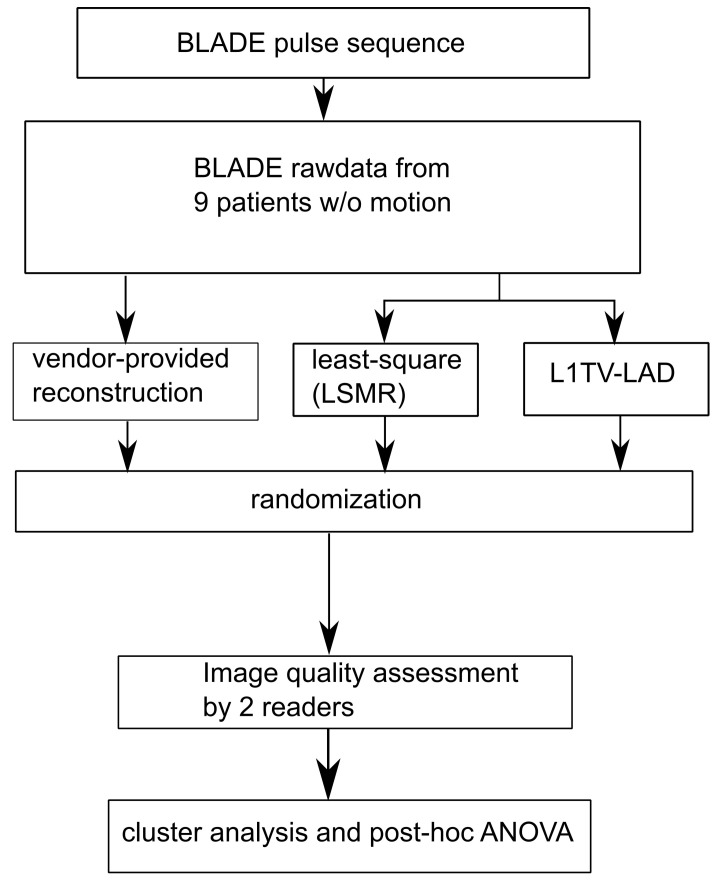
Experimental design in the study. 9 patients were scanned by the BLADE protocol. Images were generated by vendor-provided reconstruction (the sampling density compensation), the least-squares method (using LSMR) and L1TV-LAD. Images were randomized for blinded image quality assessment by 2 readers. The results were statistically analyzed by cluster analysis with repeated measures ANOVA.

The in vivo study was approved by the regional institutional review board (approval number 20-022 obtained from the Jianan Psychiatric Center, MOHW, Tainan City, Taiwan; approved on 11 September 2020; revised on 31 December 2020). Data were retrieved from patients who received head scanning for clinical indications but with negative findings, excluding patients with positive findings such as traumatic injury, tumor, stroke or infectious diseases. Patients’ genders, ages and identifiable personal information were removed after data acquisition because this minimized the risks of data breach and it is not necessary to store the information for image quality assessment. The experimental design of this study is illustrated in Figure 1. The BLADE rawdata were acquired from a 1.5 T scanner (Magneto Avanto, Syngo B17, Siemens, Erlangen, Germany). The acquisition parameters were: field-of-view (FOV) = 22–26 cm, repetition times = 4100 ms, echo times = 100 ms, slice thickness = 5 mm, matrix size = 256×256–320×320, blade size = 512×35–640×35, 2 × SENSE factor, number of blades = 14, 22–24 slices. The T2-weighted BLADE rawdata were retrieved from the Siemens 3D MRI scanner, which were then parsed using open-source twixtools [38].

### 2.3. Image Reconstruction Based on Tensor Solvers

The computer simulation tested the single-coil image reconstruction to evaluate the numerical performance of different algorithms, using a sagittal T1-weighted head MRI as a ground truth image. The k-space (Fourier domain) data were generated from a high-resolution T1 weighted turbo-spin echo sagittal brain MRI using the Python non-uniform fast Fourier transform (PyNUFFT) package [35]. Images were reconstructed using three algorithms: (1) the Sparse Equations and Least Squares (LSMR) algorithm [39] with 100 iterations at a satisfactory convergence, (2) L1TV-OLS, and (3) L1TV-LAD. The LSMR algorithm was chosen for its stability to non-symmetric matrices [39]. We reproduce the stair-casing artifacts in images by L1TV-OLS, but not in images by L1TV-LAD or LSMR.

The coil sensitivity profiles were computed from the center of k-space. The RSS method divides the complex-valued low-resolution images by the root-sum-squared of all channels. The power iteration implements the method in [40], which first smoothed the coil images by the SPIRiT kernel (ACS = 16 with 16 overlapping squares) and then applied power iterations to calculate the eigenvectors. The tensor iterations compute the coil sensitivities with the equation outlined in Equation (A14) (Appendix B).

We applied the above coil sensitivities generated by RSS, power iteration, and tensor iteration to image reconstruction. The parameters of LSMR were 100 iterations without a damping factor because applying a damping factor (the L2 regularization) on the image incurs the risk of over-suppressing the components with high noise levels. The L1TV-LAD were based on Equation (A15) using the split-Bregman method [41] and LAD data fidelity (Appendix C). The parameters of L1TV-LAD were: μ=1, λ= 0.4–1.0 (μ: the weighting of the data fidelity; λ: the regularization strength. These parameters were determined from previous literature and the pilot study). The reconstructed gray-scale images were converted to Digital Imaging and Communications in Medicine (DICOM) format without image compression.

In vivo BLADE rawdata allow parallel imaging reconstruction to be performed. The tensor iterations are performed around the center of the k-space trajectory. This is simply performed by using the NUFFT and then multiplying the data with the sampling density compensation function with the mask function, and an adjoint operation of the NUFFT. The eigenvector of each voxel was normalized to the L2 norm along the coil dimension.

We performed 100 iterations for LSMR and the L1TVLAD algorithms. The LSMR algorithm uses the hybrid CPU-GPU computing method and the GPU acceleration component is encapsulated in a PROPELLER2D_gpu class. The L1TV-LAD algorithms performed all the iterations on the accelerator with minimal data transfer between GPU and the host. A rawdata set of 22 slices and 6 coils was reconstructed within 1 min of computation times by 4 GPUs (Titan X Pascal video card (NVIDIA Corporation, Santa Clara, CA, USA) with 3584 cores, 1417 MHz–1531 MHz, 12 GB 384-bit GDDR5X memory). The reconstruction time of the vendor-provided algorithm was completed after data acquisition without delays, making a post-acquisition evaluation impossible, though an in-house developed sampling density compensation algorithm can compute the result within 5 s on a single CPU core.

### 2.4. Radiologists’ Evaluation

Two board-certified radiologists (Reader A with 8 years of clinical experience, and Reader B, with 9 years of clinical experience) separately evaluated the reconstructed MRI DICOM series with viewing software (G3 PACS viewer, INFINITT, Seoul, Korea) and DICOM-compliant monitors (model CCL354i2, TOTOKU, Tokyo, Japan), allowing readers to adjust the imaging window, level or zoom. The reconstruction algorithms were hidden from the radiologists, and they could not identify any algorithms based on the order of their appearance. To randomize the images, all the DICOM series and the vendor-provided T2 BLADE DICOM series were anonymized and were given random Service-Object Pair (SOP) Instance Unique Identifier (UID) Attributes. Therefore, the radiologists could not have been influenced by the order in which the data appeared. Due to the limited capacity to exhaust all the regularization methods, the L1TV-OLS reconstruction method was excluded from the clinical evaluation because of the obvious stair-casing and oil-painting artifacts noted in the pilot study and the literature. The method of scoring in the literature was modified [42] but we adjusted the scoring system to reflect the subtle changes in the images imposed by the different methods of reconstruction.

Absolute image scores (Table 1) were evaluated in terms of overall image quality, level of noise, tissue contrast, sharpness, and artifacts, with scores ranging from 1 (non-diagnostic) to 5 (excellent). The absolute image scores provide a subjective evaluation of the image quality from the perspective of clinical diagnosis. Scores above 3 were diagnostic, and higher scores (≥4) provided good or excellent image quality in the tests. For the relative image scores (Table 2), we assessed 4 reconstruction algorithms together with the vendor-provided T2 BLADE images, ranging from 1 (much inferior) to 5 (much better). Relative scoring used the vendor-provided image as the reference image. A score of ≥4 was interpreted as better than the vendor-provided image. Both radiologists rated the vendor-provided image as 3, because they were able to identify the vendor-provided image identical to the reference image.

The rationale of the experimental design (the absolute and the relative scores) was that, while the relative image scores gave a detailed evaluation of the subtle differences in image quality, these subtle visual differences could also be reflected in clinical values. Thus, readers were allowed to quantify the visual image quality and the perceived clinical values separately. Finally, two scores were used to represent separately the diagnostic ability and the difference in visual perception. Inter-rater reliability and repeated measure analysis of variance (ANOVA) were used to compare the scores given by the two readers.

## 3. Results

### 3.1. Coil Sensitivity Profiles

**Figure 2:**
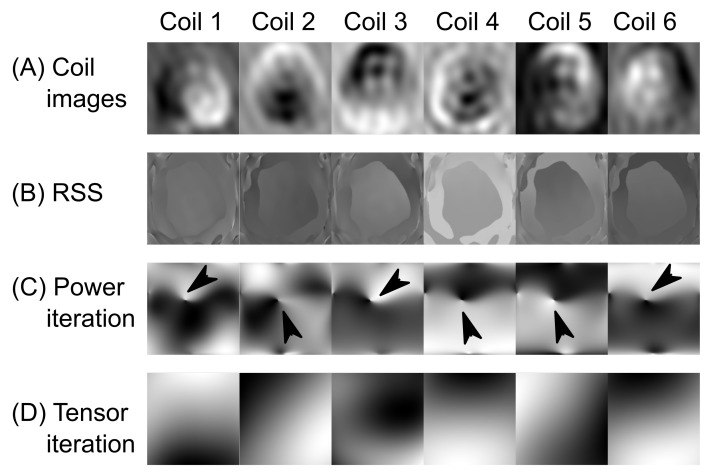
Comparisons between estimation methods for coil sensitivity profiles of coils 1–6. Only real-valued images are shown. (**A**) Coil images: The real part of the low-resolution coil images. (**B**) RSS (root-sum-squared): The low-resolution images divided by the RSS, but exhibiting phase wrapping. (**C**) The power iteration causes phase singularity (arrow heads) in all six channels. (**D**) Tensor iterations extracted smooth sensitivities. Although the intensity distributions between (**C**) and (**D**) looked different, both solutions were valid eigenvectors which were non-unique. However, the phase singularities in (**C**) could affect the homogeneity of complex-valued imagery.

Figure 2 shows that the RSS method demonstrates spatially abrupt changes in the complex components. The power iterations method led to discontinuous complex-valued components and phase singularity. The tensor iteration generated smooth coil sensitivities without abrupt discontinuous complex components or phase singularity.

**Figure 3:**
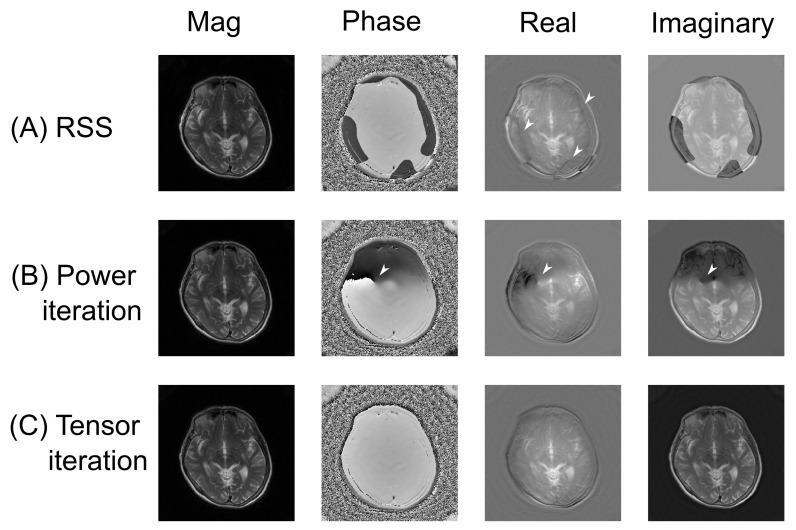
In vivo study for the least-squares reconstructed images using different coil sensitivity estimation methods. White arrow heads indicate the phase singularities in the phase image, which led to local changes of intensity in the real and the imaginary components; (**A**) RSS: images reconstructed by RSS coil sensitivities; (**B**) Power iteration: images reconstructed by the coil sensitivities of the power iterations, which were influenced by phase singularities; (**C**) Tensor iteration: images reconstructed by the coil sensitivities of the tensor iterations, which were more homogeneous than the results of (**B**).

As shown in Figure 3, the complex imagery was influenced by different coil estimation methods. All three coil sensitivity estimation methods produced final images of similar magnitude. However, the phase images and complex valued images varied with the three coil estimation methods. The RSS method generated phase wrapping in the intracranial regions, which led to local signal suppression in the real component of the image. Power iteration produced focal phase singularity, and abrupt signal changing around the thalamus, which may have affected the clinical diagnosis if a phase image was required. The tensor iteration was immune from the spatial changes. The phase image and the complex valued image parts seemed to be more homogeneous than the other two methods.

### 3.2. Numerical Evaluation of the Image Quality

**Figure 4:**
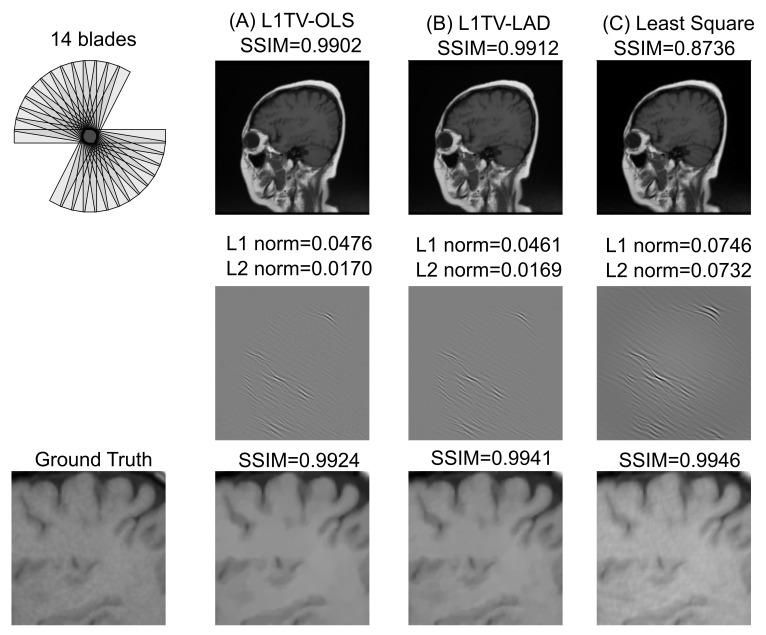
Simulated k-space of a sagittal T1-weighted TSE head MRI and reconstruction results of 3 algorithms. The k-space has 14 blades (70% coverage of the fully sampled k-space), which can occur when 30% of the blades are corrupted due to patient movement. Simulated data are reconstructed by three algorithms (**A**) the L1TV-OLS, (**B**) the L1TV-LAD, and (**C**) the least squares. Overall, L1TV-LAD achieves lower L1/L2 norm than the L1TV-OLS or least-squares, without stair-casing artifacts or cartoon-like over-smoothed image quality. Least-squares causes a higher L1/L2 norm and obvious artifacts in the difference map. The zoom-in of the imagery by 3 algorithms shows similar SSIM values (SSIM = 0.9924–0.9946).

Because the approved ethical protocols in the clinical study did not require patients to move their heads, a simulation study was performed to simulate the case of partial k-space coverage with missing blades. In this simulation, a partial k-space (14 blades out of 20, equivalent to 70% blade coverage) was generated from a T1 weighted sagittal brain MRI. Figure 4 shows the image quality metrics of a simulated brain MRI of L1TV-OLS, L1TV-LAD, and least-squares reconstructions. The full images show that L1TV-LAD was better than L1TV-OLS and least square. However, zoom-ins show that no consistent trend can be seen in the L1, L2 and SSIM image quality metrics in the magnified brain regions because the SSIM can be influenced by the magnification of the images. The L1/L2 norms show that the L1TV-LAD method is superior to the L1TV-OLS and least-squares. A moderate level of noise can be seen in the least-squares image and the ground truth, which justifies the higher SSIM of the least-squares method. However, the trend of SSIM is altered in the zoom-in images, in which the least-squares is superior to the L1TV-LAD and L1TV-OLS. Slight oil-painting artifacts can be seen in the L1TV-OLS, but were not observed in the L1TV-LAD. The contrast-to-noise ratios (CNRs) are listed in Table 3. While the least-squares method lowers the CNR, L1TV-LAD increases the overall CNR to different levels.

### 3.3. Clinical Evaluation of the Image Quality

**Figure 5:**
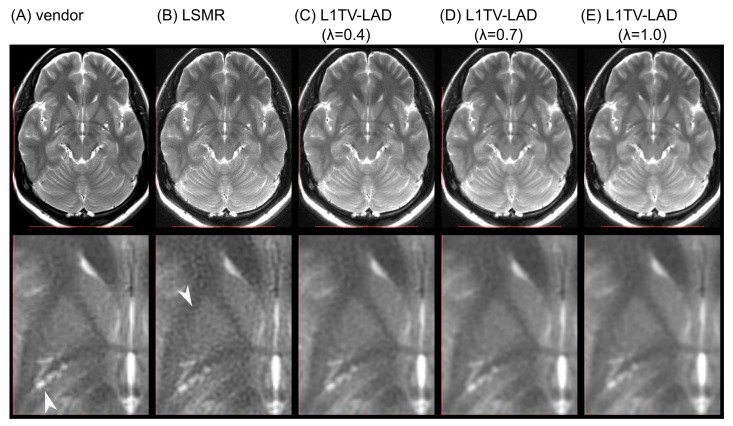
DICOM images for evaluation by radiologists. (**A**) is the vendor-provided image. (**B**) is the LSMR reconstruction. (**C**–**E**) are L1TV-LAD reconstructed images with λ = 0.4, 0.7, and 1.0, respectively. The vendor-provided image shows some zig-zag appearance (white arrow head). The LSMR reconstruction causes a higher level of noise (white arrow head). The vendor-provided image and least-squares reconstruction have some signs of the Gibbs artifact near the skull inner table and the falx.

**Figure 6:**
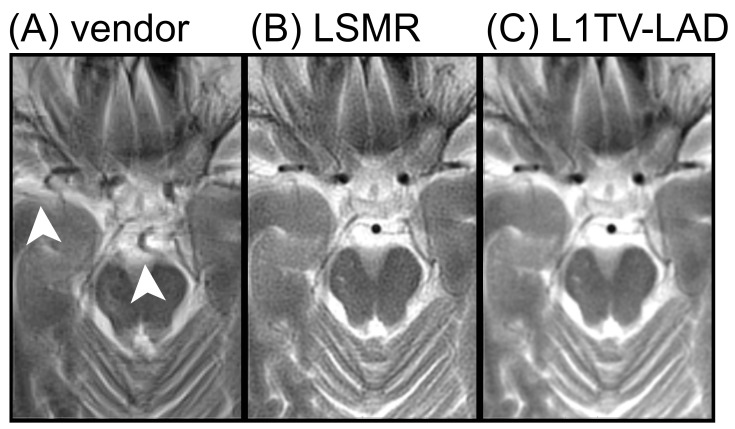
BLADE MRI with cooperative patients. (**A**) Erroneous correction for motion in the vendor-provided reconstruction, where the basilar artery is distorted and brain structures are overshadowed by artifacts (arrow heads). (**B**) Least-squares reconstruction without correcting motion. (**C**) L1TV-LAD reconstruction without correcting motion shows smooth imagery with a lower level of noise.

Nearly all five BLADE MRI imaging sets show good clinical diagnostic potential except for subtle differences in the zoom-ins (Figure 5). However, we noted in at least one slice that the vendor-provided reconstruction method yields erroneous motion correction (see Figure 6). This erroneous motion correction is a rare condition, which was not identified by operators and was impossible to correct during data acquisition.

**Figure 7:**
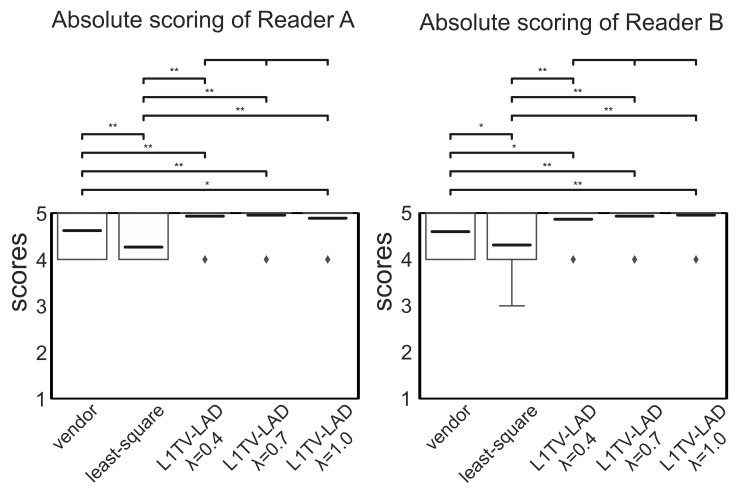
Boxplots of the absolute scores by two readers. box: the first and the third quantile, bold lines: mean, whisker: maximum and minimum (may overlapped with the box in most cases). Medians are overlapped by the box. ⧫: outliers. * p<0.01, ** p<0.001.

**Figure 8:**
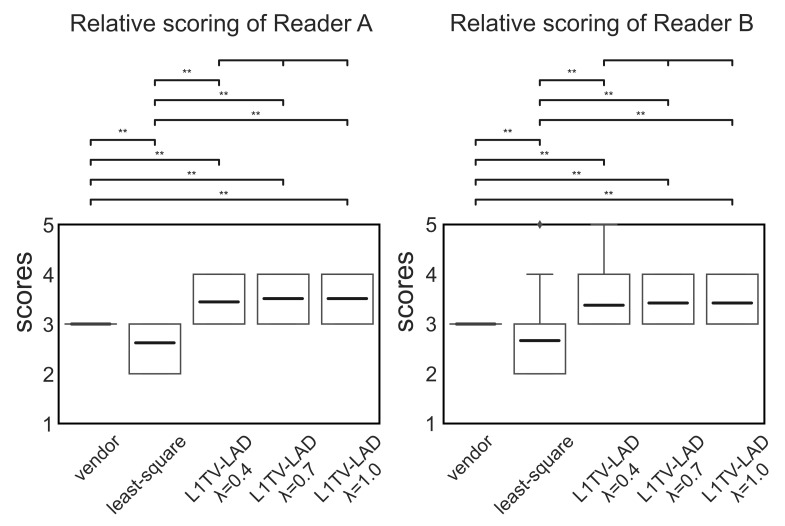
Boxplots of the relative scores by two readers. box: the first and the third quantile, bold lines: mean, whisker: maximum and minimum (may overlap with the box in most cases). Medians are overlapped by the box. ⧫: outliers. ** p<0.001.

In Figure 7 and Figure 8, the absolute and relative scores given by the two readers show high inter-rater reliability (intraclass correlation coefficient (ICC): 0.845, 95% confidence interval: 0.817, 0.87 by R language with the irr library). Analyses using repeated measures ANOVA reveal that the scores across the five methods were statistically significant (*p*-value < 0.001) in the absolute and relative scores rated by two readers. Cluster analysis using *post hoc* pairwise ANOVA shows that the scores with the least-squares method were significantly lower than those with the vendor-provided image (p<0.001 in the absolute and relative scores from Reader A, and that p<0.01 in the absolute scores and p<0.001 in the relative scores from Reader B). The scores of L1TV-LAD were significantly higher than the vendor-provided image (p<0.001 in the absolute and relative scores from the two readers) and the least-squares method (*p*-value ranged from <0.01 to *p* < 0.001) in the absolute and relative scores. Insignificant differences between the different regularization strength values of L1TV-LAD (λ=0.4,0.7,1.0) were also observed (p>0.1). The L1TV-LAD method exhibited less noise and fewer artifacts than the vendor-provided image and least-squares method (Figure 5), and was sharper and better in overall imaging quality. There is no difference of tissue contrast with any of the reconstruction methods.

## 4. Discussion

This study confirmed that L1TV-LAD improves the image quality of T2 weighted BLADE MRI in a clinical setting. The improvement is likely due to the change of data fidelity terms from the OLS to the LAD. Our finding is consistent with previous results and the recent robust compressed sensing method in computer vision [43], in which the introduction of the robust data fidelity improved the image quality even for the TV regularization. Hence, this study suggests that robust statistics may be considered for BLADE MRI, without resorting to more dedicated regularization methods.

This patient study was limited by the clinical research protocol, which does not allow intervention or impose any additional risks to patients. First, due to the nature of the inverse problems, no ground-truth images available in a clinical MRI scanning, and calculating L1/L2 norms and the SSIM were not possible on patient data. Hence, the simulation-based approach was included in this study. On the one hand, the simulation-based numerical image quality metrics provide ground-truth images, and standard numerical metrics, such as L1/L2 norms and the SSIM, can be used for evaluation. However, the numerical results can be influenced by different levels of zoom-in and noise, and other variants of SSIM [44,45] have been developed. Still, a simulation-based study is also limited because it might not accurately emulate the coil sensitivity profiles. On the other hand, a radiologist’s assessment of the absolute and relative image scoring can be a useful tool for evaluating a reconstruction method without ground-truth images, although no standard numerical metrics can be used because of the lack of ground-truth images.

Second, the raw data were collected from cooperative patients, and motion artifacts were not noticeable. We have simulated the condition of 30% missing-BLADE rejection. However, this simulation may be an inadequate approach to studying the motion artifacts. For uncooperative patients, motion artifacts have been well solved [1]. Therefore, the current evidence suggests that if patients are uncooperative, the vendor-provided motion correction algorithm can reliably restore the images. Without motion artifacts, L1TV-LAD may be used to improve the static BLADE MRI. Since switching between two reconstruction algorithms does not require a second scanning, this limitation need not prolong the total scanning time of a patient in a clinical setting.

Third, the algorithm was robust to a wide range of regularization strengths (λ = 0.4–1.0), which may well reconstruct BLADE with different thicknesses, noise-levels, or numbers of blades. However, this speculation requires future study to confirm its validity. In addition, we have not applied the algorithm to acquisition methods beyond the BLADE MRI, where readers could seek the compressed sensing MRI [46] using the iterative L1 regularization [47] when images are sparse. We have not compared compressed sensing with the L1TV-LAD for BLADE MRI. According to the Dohono-Tanner phase diagram [48,49], the compressed sensing algorithm may not reconstruct BLADE MRI faithfully because it comprises a high ratio of non-zeros in the data and images. In this non-sparse scenario, L1TV-LAD could be used instead.

Last, the pilot research collected patient data without positive findings, which precluded severe brain conditions, such as traumatic brain injuries, tumor, post-surgery, meaning that sensitivity, specificity, and the area-under-curve (AUC) of the receiver operating characteristic (ROC) curve of the disease could not be measured in this study. Although we did not explore the influence of patient demographics (such as age, gender, disease, etc.), the principle of using BLADE MRI remains unchanged regardless of patient demographics and diseases. It is likely that similar reconstruction parameters could apply to most patient groups. In future studies, it will be essential to collect data on specific diseases with positive MRI findings, from which statistical parameters can be derived.

Future study involves evaluating statistical measures, such as sensitivity, specificity, the AUC/ROC curve for brain diseases and other locations. Since PROPELLER MRI has been used in musculoskeletal imaging, liver imaging, lung imaging and body imaging, this algorithm may enhance the image quality of static BLADE MRI. Moreover, subtle pathology may be better identified without being overshadowed by noise. Because the reconstruction apparatus is a separate module outside the pulse sequence, the T2 FLuid Attenuated Inversion Recovery (T2-FLAIR) can be developed further. Beyond the iterative reconstruction method, image reconstruction may be accelerated by leveraging recent machine learning frameworks [50]. Nonetheless, radiologists should further evaluate image quality following the integration of software frameworks whose different implementations may greatly influence the image quality, causing perceptible visual changes and degraded diagnostic values.

## 5. Conclusions

We demonstrated the image quality of the L1TV-LAD algorithm for BLADE MRI is better than those of the vendor-provided image and the least-squares, but both readers reported that the noisy images were still of diagnostic value.

## Figures and Tables

**Table 1:** Absolute image scores (1: worst, 5: best).

Score	Overall Image Quality	Noise (SNR)	Tissue Contrast *	Sharpness	Artifacts ${}^{†}$
1	Non-diagnostic	All structures are noisy	Not all tissues can be separated	No structures are sharp	Severe
2	Limited	Most structures are noisy	Few tissues can be separated clearly	Few structures are sharp	Moderate
3	Diagnostic	Several structures are noisy	Several structures can be separated clearly	Several structures are sharp	Mild
4	Good	A few structures are noisy	Most structures can be separated clearly	Most structures are sharp	Minimal
5	Excellent	No noticeable noise on any image	All tissues can be separated clearly	All structures are sharp	None

*: gray matter, white matter, cerebrospinal fluid, lesion; ${}^{†}$: motion, aliasing, Gibbs, star-like artifacts.

**Table 2:** Relative image scores: as compared with the vendor-provded T2 BLADE images (1: worst, 5: best).

Score	Overall Image Quality	Noise (SNR)	Tissue Contrast *	Sharpness	Artifacts ${}^{†}$
1	Much inferior	Much inferior	Much inferior	Much inferior	Much more
2	Somewhat inferior	Somewhat inferior	Somewhat inferior	Somewhat inferior	Somewhat more
3	No distinction	No distinction	No distinction	No distinction	No distinction
4	Somewhat better	Somewhat better	Somewhat better	Somewhat better	Somewhat fewer
5	Much better	Much better	Much better	Much better	Much fewer

*: gray matter, white matter, cerebrospinal fluid, lesion; ${}^{†}$: motion, aliasing, Gibbs, star-like artifacts.

**Table 3:** Contrast-to-noise ratios (CNRs).

CNR	Vendor	Least-Square	L1TV-LAD (λ=0.4)	L1TV-LAD (λ=0.7)	L1TV-LAD (λ=1.0)
GM	55.0±14.8	48.7±8.2	75.1±11.9	89.4±14.2	101.3±16.3
WM	38.5±10.0	31.5±7.1	48.5±7.9	57.6±8.2	65.3±8.7
CSF	137.0±24.9	111.4±31.0	169.4±36.0	201.1±40.5	223.7±48.0
Thalamus	44.7±10.2	37.3±9.6	57.3±11.1	68.2±12.1	77.2±13.1
GP	36.5±8.3	28.8±7.1	44.6±7.8	53.1±8.4	60.2±9.1
CN	50.8±9.8	38.1±8.1	58.6±8.6	69.7±9.1	78.9±10.0
Putamen	44.8±9.2	35.2±8.5	54.2±9.3	66.7±20.8	73.1±10.6

GM: gray matter; WM: white matter; CSF: cerebrospinal fluid; GP: globus pallidum; CN: caudate nucleus.

**Figure A1:**
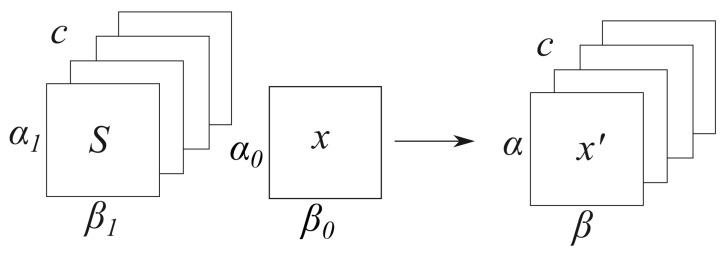
Parallel imaging shown as the index tensor operators. $x^{\alpha_{0}\beta_{0}}$ is the spatial distribution of the transverse magnetization for the object. $S^{\alpha_{1}\beta_{1}c}$ is a 3rd tensor for coil sensitivity profiles. $\alpha ,\alpha_{i},i=0,1$ indicates the first spatial dimension. $\beta ,\beta_{i},i=0,1$ indicates the second spatial dimension. *c* is the dimension for coil.

## Data Availability

The data are not publicly available due to privacy.

## Abbreviations

The following abbreviations are used in this manuscript:

ACS	Auto-Calibration Signal
ANOVA	Analysis of variance
CNR	Contrast-to-noise ratio
DICOM	Digital Imaging and Communications in Medicine
ESPIRiT	Eigenvalue Approach to Autocalibrating Parallel MRI approach
ICC	Intraclass correlation coefficient
LAD	Least absolute deviation
MRI	Magnetic resonance imaging
OLS	Ordinary least squares
PROPELLER	Periodically Rotated Overlapping ParallEL Lines with Enhanced Reconstruction
RSS	Root-sum squared
SENSE	SENSitivity Encoding
SSIM	Structural Similarity
T2 FLAIR	T2 FLuid Attenuated Inversion Recovery

## Appendix A. Parallel MRI Encoding Using the Indexed Tensor Notation

Einstein’s summation convention [51] is the mathematical notation which is used to describe multi-dimensional geometry. The index tensor notation provides a tool for annotating multi-dimensional operators beyond common second order tensors, i.e., matrices. Index notation was previously found useful in diffusion MRI for simultaneously considering the position and orientation [52]. In this study, we used indexed tensor notation to clearly identify different spatial and coil dimensions without risk of confusion.

The primary rule of tensor contraction is to contract identical Greek alphabets which simultaneously appear in two tensors as superscripts and subscripts. The superscripts and subscripts unambiguously annotate the multi-dimensional tensor operators. For example, a matrix-vector product is represented as the following index tensor notation:

(A1) $$\begin{array}{c} y^{\varsigma }\leftarrow A_{\rho }^{\varsigma }x^{\rho } \end{array}$$

The contravariant ς is the first dimension (row) of matrix *A*. The covariant ρ is the second dimension (column) of matrix *A*. This representation is equivalent to the element-wise matrix-vector multiplications of Ax=y:

(A2) $$\begin{array}{c} y\left(\varsigma \right)=\sum_{\rho =1}^{P}A\left(\varsigma ,\rho \right)\cdot x\left(\rho \right),\;\rho =1,2,\cdots ,P \end{array}$$

A introduction to Einstein notation can be found in the materials of tensor calculus [53].

The index tensor notation for 2D fast Fourier transform reads as follows:

(A3) $$\begin{array}{c} y^{\kappa \lambda }\leftarrow F1_{\alpha }^{\kappa }F2_{\beta }^{\lambda }x^{\alpha \beta } \end{array}$$

$F1_{\alpha }^{\kappa }F2_{\beta }^{\lambda }=F_{\alpha \beta }^{\kappa \lambda }$ is the 2D Fourier transform. The meaning of this representation is that the 2D image $x^{\alpha \beta }$ is transformed by $F1_{\alpha }^{\kappa }$ along the first axis (normally known as the *x*-axis) and the second axis by $F2_{\beta }^{\lambda }$ (normally known as the *y*-axis). The k-space is spanned by the κ,λ axes, which are normally denoted as the kx,ky in the MRI community

Extending the Cartesian k-space to non-Cartesian is straightforward. By reformatting the spatial encoding as tensor operators, the non-Cartesian Fourier transform is well described as the interpolation in k-space:

(A4) $$\begin{array}{c} y^{\mu }\leftarrow \delta_{\mu_{1}\mu_{2}}^{\mu }{V_{2}}_{\lambda }^{\mu_{2}}{V_{1}}_{\kappa }^{\mu_{1}}F_{\alpha \beta }^{\kappa \lambda }x^{\alpha \beta } \end{array}$$

in which the Kronecker delta is defined as:

(A5) $$\delta_{\mu_{1}\mu_{2}}^{\mu }=\left\{\begin{array}{cc} 1, & \;if\;\mu =\mu_{1}=\mu_{2}\\ 0, & otherwise \end{array}\right.$$

$y^{\mu }$ is the 1D non-Cartesian data. $F_{\alpha \beta }^{\kappa \lambda }$ is the 2D Fourier transform. $\delta_{\mu_{1}\mu_{2}}^{\mu }{V_{2}}_{\lambda }^{\mu_{2}}{V_{1}}_{\kappa }^{\mu_{1}}$ indicates the tensor operators for interpolating grid data to the off-grid coordinates. The current notation looks different from the previous Einstein’s notation introduced in the tensor conjugate gradient method [54], in which a single high-order tensor represents the multi-dimensional interpolation.

To elucidate the similarity between the current separated tensors and the previous representation we define $V^{\prime }$:

(A6) $$\begin{array}{c} {V^{\prime }}_{\kappa \lambda }^{\mu }=\delta_{\mu_{1}\mu_{2}}^{\mu }{V_{2}}_{\lambda }^{\mu_{2}}{V_{1}}_{\kappa }^{\mu_{1}} \end{array}$$

${V^{\prime }}_{\kappa \lambda }^{\mu }$ represents the 2D interpolator as a single higher-order tensor, which is similar to the notation in [54].

The index tensor operators of the general non-Cartesian k-space can be further decomposed if the k-space acquisition has shown certain periodicity in the acquisition order. The PROPELLER MRI [1] oversamples the center of the k-space and rotational or translational motion compensations can be made before the final reconstruction. Each BLADE is a rotated Cartesian k-space. The tensor operator for PROPELLER is as follows:

(A7) $$y^{\mu \nu \theta c}\leftarrow \delta_{\theta_{1}\theta_{2}}^{\theta }\delta_{\mu_{1}\mu_{2}}^{\mu }\delta_{\nu_{1}\nu_{2}}^{\nu }E1_{\alpha }^{\mu_{1}\nu_{1}\theta_{1}}E2_{\beta }^{\mu_{2}\nu_{2}\theta_{2}}\delta_{\beta_{0}\beta_{1}}^{\beta }\delta_{\alpha_{0}\alpha_{1}}^{\alpha }S^{\alpha_{1}\beta_{1}c}x^{\alpha_{0}\beta_{0}}$$

where $E1_{\alpha }^{\mu_{1}\nu_{1}\theta_{1}}$ and $E2_{\beta }^{\mu_{2}\nu_{2}\theta_{2}}$ are fourth-order tensors, indicating the tensor encoding operators for the x,y axes, respectively. $\delta_{\theta_{1}\theta_{2}}^{\theta }$, $\delta_{\mu_{1}\mu_{2}}^{\mu }$ and $\delta_{\nu_{1}\nu_{2}}^{\nu }$ are Kronecker delta functions to select the “coincidence” when $\mu =\mu_{1}=\mu_{2}$, $\nu =\nu_{1}=\nu_{2}$ and $\theta =\theta_{1}=\theta_{2}$. $y^{\mu \nu \theta c}$ indicates the data of the *c*-th coil, which are acquired at angle θ, phase encoding distance ν and radial distance μ.

(A8) $$\begin{array}{c} E1_{\alpha }^{\mu_{1}\nu_{1}\theta_{1}}=exp\left[-2\pi j\left\{\frac{\mu_{1}-M_{c}}{M_{max}}cos\theta_{1}-\frac{\nu_{1}-L_{c}}{M_{max}}sin\theta_{1}\right\}\left(\alpha -\alpha_{c}\right)\right]\\ E2_{\beta }^{\mu_{2}\nu_{2}\theta_{2}}=exp\left[-2\pi j\left\{\frac{\mu_{2}-M_{c}}{M_{max}}cos\theta_{2}+\frac{\nu_{2}-L_{c}}{M_{max}}cos\theta_{2}\right\}\left(\beta -\beta_{c}\right)\right] \end{array}$$

$\alpha_{c},\beta_{c}$ are the center of the 2D spatial dimensions, $M_{c},L_{c}$ are the center for the frequency and the phase encoding directions.

The operators E1 and E2 can be further decomposed into sub-tensor operators:

(A9) $$\begin{array}{c} E1_{\alpha }^{\mu_{1}\nu_{1}\theta_{1}}=\delta_{\theta_{1}^{\prime }{\theta^{\prime \prime }}_{1}}^{\theta_{1}}\delta_{\alpha }^{\alpha^{\prime }\alpha^{\prime \prime }}E1_{\alpha^{\prime \prime }}^{\mu_{1}\theta_{1}^{\prime }}E{1^{\prime \prime }}_{\alpha^{\prime \prime }}^{\nu_{1}{\theta^{\prime \prime }}_{1}}\\ E2_{\beta }^{\mu_{2}\nu_{2}\theta_{2}}=\delta_{\theta_{2}^{\prime }{\theta^{\prime \prime }}_{2}}^{\theta_{2}}\delta_{\beta }^{\beta^{\prime }\beta^{\prime \prime }}E2_{\beta^{\prime \prime }}^{\mu_{2}\theta_{2}^{\prime }}E{2^{\prime \prime }}_{\beta^{\prime \prime }}^{\nu_{2}{\theta^{\prime \prime }}_{2}} \end{array}$$

which shows that the *x*-*y* directional tensors are composed of the frequency-encoding tensor (as in radial MRI) and the phase-encoding tensor.

## Appendix B. Parallel Imaging Reconstruction

Sensitivity Encoding (SENSE) [55] acquires low-resolution images from prescans or self-calibrating k-space, and using Eigenvalue Approach to Autocalibrating Parallel MRI approach (ESPIRiT) [40] is a popular method of extracting the coil sensitivity profiles from the coil images. In ESPIRiT, the coil sensitivities were solved by moving the auto-calibrating signal (ACS) windows in k-space, and the right singular vectors are reformatted as the smooth bases of the coil images. The ESPIRiT method solves the eigenvector on top of the images smoothed by SPIRiT kernels, which perform well for magnitude imagery. However, the eigenvectors are non-unique and the numerical instabilities can cause phase variations. Recently, other modified coil estimation methods [28,36] have been proposed. GRAPPA type non-Cartesian k-space has also been proposed [56].

The index tensor form allows the eigendecomposition to be reliably estimated, since a 2D image tensor $x^{\alpha_{0}\beta_{0}}$ to be multiplied by the coil sensitivity profile can be written as a 3rd tensor $S^{\alpha_{1}\beta_{1}c}$:

(A10) $${x^{\prime }}^{\alpha \beta c}\leftarrow \delta_{\beta_{0}\beta_{1}}^{\beta }\delta_{\alpha_{0}\alpha_{1}}^{\alpha }S^{\alpha_{1}\beta_{1}c}x^{\alpha_{0}\beta_{0}}$$

as illustrated in Figure A1.

The index notation is well adapted to multislice parallel imaging reconstruction. Consider the encoding tensor operator for multislice 2D PROPELLER MRI:

(A11) $$\begin{array}{c} y^{\mu \nu \theta \gamma^{\prime }c}\leftarrow M_{\alpha \beta \gamma }^{\mu \nu \theta \gamma^{\prime }c}x^{\alpha \beta \gamma } \end{array}$$

μ is the radial distance, ν is the phase encoding direction, θ is the angle, *c* is the coil dimension, γ and $\gamma^{\prime }$ indicate the slice dimension. $M_{\alpha \beta \gamma }^{\mu \nu \theta \gamma^{\prime }c}$ consists of non-Cartesian Fourier encoding and multislice coil sensitivities.

Calibrating the coil sensitivities requires a subtensor ${M_{sub}}_{\alpha \beta }^{\mu^{\prime }\nu^{\prime }\theta^{\prime }}$ which performs 2D encoding around the center of k-space (as ACS in ESPIRiT). A pseudo-inverse operator ${M_{sub}}_{\mu^{\prime }\nu^{\prime }\theta^{\prime }}^{†\alpha \beta }$ indicates the reconstruction process which generates low-resolution coil images:

(A12) $$\begin{array}{c} z^{\alpha \beta \gamma^{\prime }c}\leftarrow {M_{sub}}_{\mu^{\prime }\nu^{\prime }\theta^{\prime }}^{†\alpha \beta }{\left\{y_{sub}\right\}}^{\mu^{\prime }\nu^{\prime }\theta^{\prime }\gamma^{\prime }c} \end{array}$$

${\left\{y_{sub}\right\}}^{\mu^{\prime }\nu^{\prime }\theta^{\prime }\gamma^{\prime }c}$ is the Cartesian or non-Cartesian rawdata in the k-space, $z^{\alpha \beta \gamma^{\prime }c}$ is the smoothed coil images. ${M_{sub}}_{\mu^{\prime }\nu^{\prime }\theta^{\prime }}^{†\alpha \beta }$ can be approximated by a direct or iterative solver, e.g., the sampling density compensation method, iterative least-squares solvers or singular-value decomposition.

Equipped with the pseudo-inverse operator, the power iteration computes the coil sensitivity profile $S^{\alpha_{0}\beta_{0}\gamma_{0}c_{0}}$ by evaluating the lower resolution image $z_{\alpha_{2}\beta_{2}\gamma_{2}^{\prime }}^{c}$ in every iteration:

(A13) $$\begin{array}{cc} Given\; & S{\left(0\right)}^{\alpha_{0}\beta_{0}\gamma_{0}c_{0}},\;for\;k=0,1,\cdots ,\;iterate\;until\;convergence\\ \; & S{\left(k\right)}^{\prime \alpha \beta \gamma c}\leftarrow \delta_{\alpha_{0}\alpha_{1}}^{\alpha \alpha_{2}}\delta_{\beta_{0}\beta_{1}}^{\beta \beta_{2}}\delta_{\gamma_{0}\gamma_{1}^{\prime }}^{\gamma \gamma_{2}^{\prime }}z_{\alpha_{2}\beta_{2}\gamma_{2}^{\prime }}^{c}{\bar{z}}_{c_{0}}^{\alpha_{1}\beta_{1}\gamma_{1}^{\prime }}S{\left(k\right)}^{\alpha_{0}\beta_{0}\gamma_{0}c_{0}}\\ \; & \sigma^{\alpha \beta \gamma }\leftarrow {∥S{\left(k\right)}^{\prime \alpha \beta \gamma c}∥}_{c}\\ \; & S{\left(k+1\right)}^{\alpha_{0}\beta_{0}\gamma_{0}c_{0}}\leftarrow \delta_{\alpha \alpha^{\prime }}^{\alpha_{0}}\delta_{\beta \beta^{\prime }}^{\beta_{0}}\delta_{\gamma \gamma^{\prime }}^{\gamma_{0}}S{\left(k\right)}^{{}^{\prime }\alpha \beta \gamma c_{0}}{\sigma^{-1}}^{\alpha^{\prime }\beta^{\prime }\gamma^{\prime }} \end{array}$$

where ${∥S{\left(k\right)}^{{}^{\prime }\alpha \beta \gamma c}∥}_{c}$ represents the Frobenius norm along the coil dimension, $z_{\alpha_{2}\beta_{2}\gamma_{2}^{\prime }}^{c}$ indicates the low resolution image and ${\bar{z}}_{c_{0}}^{\alpha_{1}\beta_{1}\gamma_{1}^{\prime }}$ is its conjugate. The iteration stops at a satisfactory convergence.

With power iterations for estimating coil-sensitivities in the image domain, numerical instabilities and phase fluctuations can corrupt the quality of the coil-sensitivities. Although the problem of phase singularity is minimal in magnitude images, it can severely degrade the image quality of phase images. To circumvent the phase singularity in the power iteration, one can leverage the index tensor form and replace the low resolution image by the k-space data. This approach transforms Equation (A12) into the following tensor iteration:

(A14) $$\begin{array}{cc} U_{\alpha_{0}\beta_{0}\gamma_{0}c_{0}}^{\alpha \beta \gamma c} & =\delta_{\gamma_{2}^{\prime }}^{\gamma_{1}^{\prime }}\delta_{\mu_{0}^{\prime }\mu_{2}^{\prime }}^{\mu_{1}^{\prime }\mu_{3}^{\prime }}\delta_{\nu_{0}^{\prime }\nu_{2}^{\prime }}^{\nu_{1}^{\prime }\nu_{3}^{\prime }}\delta_{\theta_{0}^{\prime }\theta_{2}^{\prime }}^{\theta_{1}^{\prime }\theta_{3}^{\prime }}{\left\{F_{sub}^{-1}D\right\}}_{\mu_{3}^{\prime }\nu_{3}^{\prime }\theta_{3}^{\prime }}^{\alpha \beta \gamma }{\left\{y_{sub}\right\}}^{\mu_{2}^{\prime }\nu_{2}^{\prime }\theta_{2}^{\prime }\gamma_{2}^{\prime }c}{\left\{{\bar{y}}_{sub}\right\}}_{\mu_{1}^{\prime }\nu_{1}^{\prime }\theta_{1}^{\prime }\gamma_{1}^{\prime }c_{0}}{\left\{DF_{sub}\right\}}_{\alpha_{0}\beta_{0}\gamma_{0}}^{\mu_{0}^{\prime }\nu_{0}^{\prime }\theta_{0}^{\prime }}\\ Given\; & S{\left(0\right)}^{\alpha_{0}\beta_{0}\gamma_{0}c_{0}},\;for\;k=0,1,\cdots ,\;iterate\;until\;convergence\\ \; & S{\left(k\right)}^{\prime \alpha \beta \gamma c}\leftarrow U_{\alpha_{0}\beta_{0}\gamma_{0}c_{0}}^{\alpha \beta \gamma c}S{\left(k\right)}^{\alpha_{0}\beta_{0}\gamma_{0}c_{0}}\\ \; & \sigma^{\alpha \beta \gamma }\leftarrow {∥S{\left(k\right)}^{\prime \alpha \beta \gamma c}∥}_{c}\\ \; & S{\left(k+1\right)}^{\alpha_{0}\beta_{0}\gamma_{0}c_{0}}\leftarrow \delta_{\alpha \alpha^{\prime }}^{\alpha_{0}}\delta_{\beta \beta^{\prime }}^{\beta_{0}}\delta_{\gamma \gamma^{\prime }}^{\gamma_{0}}S{\left(k\right)}^{\prime \alpha \beta \gamma c_{0}}{\sigma^{-1}}^{\alpha^{\prime }\beta^{\prime }\gamma^{\prime }} \end{array}$$

in which the pseudo-inverse operator ${M_{sub}}_{\mu^{\prime }\nu^{\prime }\theta^{\prime }}^{†\alpha \beta \gamma }={\left\{F_{sub}^{-1}D\right\}}_{\mu^{\prime }\nu^{\prime }\theta^{\prime }}^{\alpha \beta \gamma }$ consists of the inverse Fourier transform of the center of k-space ($F_{sub}^{-1}$) and the sampling density compensation function (*D*).

In the current approach, we find that tensor iteration appears better than ESPIRiT because the power iterations evaluate the non-Cartesian data in every iteration. Therefore, the eigenvector is stabilized by the constraint of the k-space center, which mitigates the phase singularity of power iterations in image domain. Empirical results show that the iteration converges within 20 iterations.

## Appendix C. Optimization Algorithms

The LSMR and the L1 total-variation LAD algorithms were integrated into the reconstruction pipeline to solve the least-squares problem. These two algorithms were selected based on the basis of previous visual observations, in which no stair-casing, oil-painting or blocky artifacts were noticed in the two solvers. The L1TV-LAD reconstruction involves minimizing the cost function, which consists of the data consistency term and the regularization term. A slight modification of the L1TV-LAD is that we regularized the data consistency term in the non-Cartesian Fourier domain, as opposed to the adjoint transformed images in the previous implementation L1TV regularization [34]. In single coil fast-spin echoes imaging, adjoint transformed images are all real-values, but complex values exist when coil sensitivities are considered. Furthermore, the multi-coil problem is considered together with the non-Cartesian k-space. Therefore it is advantageous to reformulate the problem into the index tensor form. Using the tensor operators, we proceeded to formulate the LAD version of the split-Bregmen method [41] for non-Cartesian k-space:

(A15) $$\begin{array}{cc} \; & K^{\iota_{1}\eta_{1}}=F_{\alpha_{1}\beta_{1}}^{\iota_{1}\eta_{1}}\left(\mu {\left(A^{H}DA\right)}_{\alpha_{0}\beta_{0}}^{\alpha_{1}\beta_{1}}-\lambda \Delta_{\alpha_{0}\beta_{0}}^{\alpha_{1}\beta_{1}}+\gamma I_{\alpha_{0}\beta_{0}}^{\alpha_{1}\beta_{1}}\right){F^{-1}}_{\iota_{0}\eta_{0}}^{\alpha_{0}\beta_{0}}I^{\iota_{0}\eta_{0}}\\ \; & Initialize:\;u{\left(0\right)}^{\alpha \beta \gamma }\;=\;{\left(A^{†}\right)}_{\mu \nu \theta c}^{\alpha \beta }f^{\mu \nu \theta \gamma c},\\ \; & \;\;\;d_{x}{\left(0\right)}^{\alpha^{\prime }\beta \gamma }=d_{y}{\left(0\right)}^{\alpha \beta^{\prime }\gamma }=b_{x}{\left(0\right)}^{\alpha^{\prime }\beta \gamma }=b_{y}{\left(0\right)}^{\alpha \beta^{\prime }\gamma }=0\\ \; & \;\;\;b_{f}{\left(0\right)}^{\mu \nu \theta \gamma c}=d_{f}{\left(0\right)}^{\mu \nu \theta \gamma c}=0\\ \; & for\;k=0,1,\cdots \;iterate\;until\;convergence:\\ \; & \;\;rhs{\left(k\right)}^{\alpha \beta \gamma }=\mu {\left(A^{†}\right)}_{\mu \nu \theta c}^{\alpha \beta }{\left(f\left(k\right)+d_{f}\left(k\right)-b_{f}\left(k\right)\right)}^{\mu \nu \theta \gamma c}+\lambda {\nabla^{T}}_{\alpha^{\prime }}^{\alpha }\left(d_{x}{\left(k\right)}^{\alpha^{\prime }\beta \gamma }-b_{x}{\left(k\right)}^{\alpha^{\prime }\beta \gamma }\right)+\\ \; & \;\;\;\;\;\;\;\;\;\;\;\;\lambda {\nabla^{T}}_{\beta^{\prime }}^{\beta }\left(d_{y}{\left(k\right)}^{\alpha \beta^{\prime }\gamma }-b_{y}{\left(k\right)}^{\alpha \beta^{\prime }\gamma }\right)\\ \; & \;\;u{\left(k+1\right)}^{\alpha \beta \gamma }={\left(F^{-1}K^{-1}F\right)}_{\alpha^{\prime }\beta^{\prime }}^{\alpha \beta }\;rhs{\left(k\right)}^{\alpha^{\prime }\beta^{\prime }\gamma }\\ \; & \;\;d_{x}{\left(k+1\right)}^{\alpha^{\prime }\beta \gamma }=max\left(s{\left(k+1\right)}^{\alpha^{\prime }\beta \gamma }-1/\lambda ,0\right)⊙\left(\nabla_{\alpha }^{\alpha^{\prime }}u{\left(k+1\right)}^{\alpha \beta \gamma }+b_{x}{\left(k\right)}^{\alpha^{\prime }\beta \gamma }\right)⊘\\ & \;\;\;\;\;\;\;\;\;\;\;\;\;\;\;\;\;\;\;\;s{\left(k+1\right)}^{\alpha^{\prime }\beta \gamma }\\ \; & \;\;d_{y}{\left(k+1\right)}^{\alpha \beta^{\prime }\gamma }=max\left(s{\left(k+1\right)}^{\alpha \beta^{\prime }\gamma }-1/\lambda ,0\right)⊙\left(\nabla_{\beta }^{\beta^{\prime }}u{\left(k+1\right)}^{\alpha \beta \gamma }+b_{y}{\left(k\right)}^{\alpha \beta^{\prime }\gamma }\right)⊘\\ & \;\;\;\;\;\;\;\;\;\;\;\;\;\;\;\;\;\;\;\;s{\left(k+1\right)}^{\alpha \beta^{\prime }\gamma }\\ \; & \;\;d_{f}{\left(k+1\right)}^{\mu \nu \theta \gamma c}=shrink\left(A_{\alpha \beta \gamma }^{\mu \nu \theta \gamma c}u{\left(k+1\right)}^{\alpha \beta \gamma }+b_{f}{\left(k\right)}^{\mu \nu \theta \gamma c},1/\mu \right)\\ \; & \;\;b_{x}{\left(k+1\right)}^{\alpha^{\prime }\beta \gamma }=b_{x}{\left(k\right)}^{\alpha^{\prime }\beta \gamma }+\left(\nabla_{\alpha }^{\alpha^{\prime }}u{\left(k+1\right)}^{\alpha \beta \gamma }-d_{x}{\left(k+1\right)}^{\alpha^{\prime }\beta \gamma }\right)\\ \; & \;\;b_{y}{\left(k+1\right)}^{\alpha \beta^{\prime }\gamma }=b_{y}{\left(k\right)}^{\alpha \beta^{\prime }\gamma }+\left(\nabla_{\beta }^{\beta^{\prime }}u{\left(k+1\right)}^{\alpha \beta \gamma }-d_{y}{\left(k+1\right)}^{\alpha \beta^{\prime }\gamma }\right)\\ \; & \;\;b_{f}{\left(k+1\right)}^{\mu \nu \theta \gamma c}=b_{f}{\left(k\right)}^{\mu \nu \theta \gamma c}+\left(A_{\alpha \beta \gamma }^{\mu \nu \theta \gamma c}u{\left(k+1\right)}^{\alpha \beta \gamma }-d_{f}{\left(k+1\right)}^{\mu \nu \theta \gamma c}\right)\\ \; & \;\;f{\left(k+1\right)}^{\mu \nu \theta \gamma c}=f{\left(k\right)}^{\mu \nu \theta \gamma c}+f^{\mu \nu \theta \gamma c}-A_{\alpha \beta }^{\mu \nu \theta c}u{\left(k+1\right)}^{\alpha \beta \gamma } \end{array}$$

Here, we used the tensor operators ⊙ and ⊘ to indicate the Hadamard product and division. The directional gradient operators of the non-curved coordinates $\nabla_{x},\nabla_{y}$ are represented as $\nabla_{\alpha }^{\alpha^{\prime }},\nabla_{\beta }^{\beta^{\prime }}$.
